# Development of bullous pemphigoid during treatment of psoriasis with ustekinumab: a case report and literature review

**DOI:** 10.3389/fmed.2023.1171802

**Published:** 2023-05-03

**Authors:** Luyang Kong, Dawei Huang, Jiajing Lu, Yuexin Zhang, Ying Li, Xuemei Yi, Yuling Shi

**Affiliations:** ^1^Department of Dermatology, Shanghai Skin Disease Hospital, Tongji University School of Medicine, Shanghai, China; ^2^Institute of Psoriasis, Tongji University School of Medicine, Shanghai, China

**Keywords:** ustekinumab, bullous pemphigoid, psoriasis, case report, treatment

## Abstract

Ustekinumab is a biological therapy that has been approved for treating moderate-to-severe psoriasis. Although injection site reactions, nasopharyngitis, headaches, and infections are the common adverse events associated with ustekinumab, the development of bullous pemphigoid (BP) is also thought to be related to ustekinumab. Given that psoriasis itself can be complicated by BP, it is worthwhile to investigate the relationship between ustekinumab, psoriasis, and BP. Here we report a case of a male patient who developed BP twice after psoriasis treatment with ustekinumab. The patient’s psoriasis and BP were brought under control by discontinuing ustekinumab and administering methotrexate, minocycline, and topical corticosteroids. Because of the increasing use of biologics in patients with psoriasis, BP should be considered a potential adverse event associated with ustekinumab.

## Introduction

Bullous pemphigoid (BP) is a chronic antibody-mediated autoimmune disease characterized by blistering. Several biological agents have been reported to trigger BP (1), including guselkumab, secukinumab, and adalimumab. Herein, we report a case of a psoriasis patient who developed BP after treatment with ustekinumab.

**FIGURE 1:**
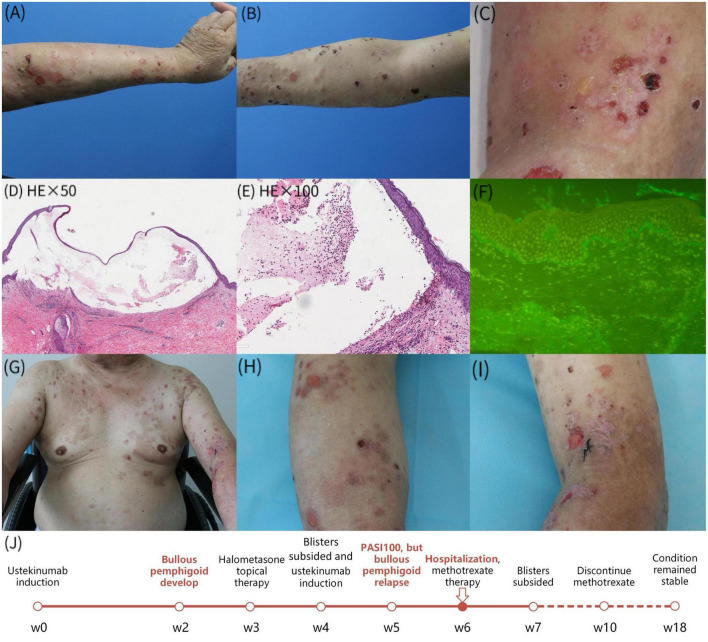
**(A–C)** The diffuse erythema, tense bullae and erosions involved both upper limbs; **(D,E)** skin biopsy revealed a subepidermal blister and the superficial dermal inflammation consisting of lymphocytes and eosnophils; **(F)** direct immunofluorescence demonstrated linear deposits of IgG along basement membrane zone; **(G–I)** improvement of skin lesions after 1 week of treatment; **(J)** the timeline of the patient’s clinical symptoms.

A 71-year-old Chinese man with pruritic blisters on his upper limbs was admitted to our hospital. He had a 7-year history of psoriasis and received a subcutaneous injection of 45 mg ustekinumab. Two weeks after the first injection, he developed blisters on his upper limbs (Figures 1A, B). The skin lesions mostly subsided after treatment with topical halometasone cream. However, the pruritic blisters on his upper limbs reappeared 1 week after the second ustekinumab injection (Figure 1C). Furthermore, the psoriasis lesions had essentially subsided. The patient had a history of hypertension, diabetes, and hemiplegia after cerebral hemorrhage and received long-term treatment with nifedipine, valsartan, hydrochlorothiazide, pioglitazone, metformin, and insulin. Laboratory tests revealed an immunoglobulin E (IgE) level and absolute blood eosinophil count of 17100 IU/ml (normal, <100 IU/ml) and 1.58 × 10^9^/L, respectively. Enzyme-linked immunosorbent assay revealed serum immunoglobulin G (IgG) against BP180 and BP230 (119.9 and 111.8 U/ml, respectively). Histologic examination showed a subepidermal blister and superficial dermal inflammation comprising lymphocytes and eosinophils (Figures 1D, E). Direct immunofluorescence showed linear IgG staining along the basement membrane zone (Figure 1F). Genetic testing of human leukocyte antigen class II alleles showed that the patient had *DQB1 02:02*, *DQB1 03:03*, and *DRB1 07:01* alleles, which have been reported to be BP-protective alleles. Therefore, a diagnosis of BP induced by ustekinumab was considered. The patient was treated with 10 mg oral methotrexate weekly, 100 mg minocycline twice daily, and topical corticosteroids. The skin lesions improved significantly after 2 weeks (Figures 1G–I). Consequently, the treatment was suspended and the patient was monitored for 2 months; his condition remained stable without new blisters or psoriasis lesions. The timeline is summarized in Figure 1J.

Biologics have become key treatments for moderate-to-severe psoriasis. However, with their increasing use, more and more cases of biologic-induced BP (BIBP) have been reported (1). The clinical, histological, and immunopathological features of BIBP are similar to idiopathic BP. When BP develops in patients prescribed biologics, it is difficult to determine whether there is a clear causal relationship between BP and biologics. Our patient developed BP after the first ustekinumab injection, and the same lesions appear after reinjection. There was no relapse during the 2-month follow-up period after ustekinumab and other therapeutic drugs were discontinued. His previous medications for hypertension and diabetes were ruled out as causes because their long-term use had not caused BP. Based on the Naranjo Adverse Reaction Probability Scale and the Karch-Lasagna algorithm, the causal relationship between BP and ustekinumab was classified as “probable.” Therefore, we believe that the BP in our patient was related to ustekinumab use.

Development of BP following the use of ustekinumab has been previously reported (Table 1) (2–6). In these five cases, the duration between the introduction of biologics and the onset of blistering diseases varied vastly, ranging from 1 month to 21 months. Biologic agents were stopped in all cases, and blisters were controlled by using topical corticosteroids in one case. In five cases including ours, systemic treatments such as corticosteroids, dapsone, methotrexate, and cyclosporine were required. Although the exact relationship remains uncertain, it is notable that there was a history of anti-TNF-a treatment in all BP cases. The mechanism may be related to ustekinumab changing the immunological state from T helper cell (Th)1 to Th2 dominance. Furthermore, ustekinumab may alter the balance of immune complex formation and clearance, leading to an accumulation of immune complexes in the skin and the development of BIBP. Moreover, our patient possessed a protective gene for BP, and therefore, there was a lower probability of him developing BP naturally compared with that in the general population. Our patient nevertheless developed BP, which was thought to be the strong effect of ustekinumab on the immune system.

**TABLE 1:** Reported cases of bullous pemphigoid occurring under treatment with ustekinumab.

	Molecular study	Prior use of biologics	Incubation period	Type 2 diabetes	Hypertension	Topical treatment	Systemic treatment
Querol-Cisneros et al. (2)	Anti-BP180 (NC16A, 120 kDa LAD-1 and C-terminal domain), BP230 negative	Adalimumab	2 months	N	N	NR	Oral corticosteroids
Marin et al. (3)	Anti-BP180NC16A negative	Adalimumab	10 months	N	N	Clobetasol, zinc sulfate 1/1000	Intravenous methylprednisolone, dapsone
Onsun et al. (4)	NR	Etanercept	1 month	Y (metformin)	N	NR	Prednisolone, cyclosporine
Nakayama et al. (5)	Anti-BP180NC16A positive	Infliximab	21 months	N	N	NR	Prednisolone
Le Guern et al. (6)	NR	Etanercept, Adalimumab	9 months	Y (metformin)	Y	Corticosteroids	N

Y, yes; N, none; NR, not reported.

## Conclusion

In conclusion, BP should be taken into account when blisters develop during the use of biologics, especially ustekinumab. However, further research is needed to fully understand BIBP.

## Data availability statement

The original contributions presented in this study are included in the article/supplementary material, further inquiries can be directed to the corresponding authors.

## Ethics statement

Written informed consent was obtained from the individual(s) for the publication of any potentially identifiable images or data included in this article.

## Author contributions

All authors listed have made a substantial, direct, and intellectual contribution to the work, and approved it for publication.
